# Peak forces and lateral resolution in amplitude modulation force microscopy in liquid

**DOI:** 10.3762/bjnano.4.96

**Published:** 2013-12-06

**Authors:** Horacio V Guzman, Ricardo Garcia

**Affiliations:** 1 Instituto de Ciencia de Materiales de Madrid, CSIC, Sor Juan Ines de la Cruz 3, 28049 Madrid, Spain

**Keywords:** force microscopy, lateral resolution, nanomechanics, peak force

## Abstract

The peak forces exerted on soft and rigid samples by a force microscope have been modeled by performing numerical simulations of the tip motion in liquid. The forces are obtained by using two contact mechanics models, Hertz and Tatara. We present a comparison between the numerical simulations and three analytical models for a wide variety of probe and operational parameters. In general, the forces derived from analytical expressions are not in good quantitative agreement with the simulations when the Young modulus and the set-point amplitude are varied. The only exception is the parametrized approximation that matches the results given by Hertz contact mechanics for soft materials and small free amplitudes. We also study the elastic deformation of the sample as a function of the imaging conditions for materials with a Young modulus between 25 MPa and 2 GPa. High lateral resolution images are predicted by using both small free amplitudes (less than 2 nm for soft materials) and high set-point amplitudes.

## Introduction

The high-resolution imaging of heterogeneous materials, in particular soft materials in liquid, by amplitude modulation atomic force microscopy (AM-AFM) is an active area of research in nanotechnology [1–11]. In AM-AFM, a sharp tip is attached at the end of a microcantilever that oscillates at or near its resonant frequency. When the tip is in close proximity to the sample, the amplitude and the phase shift of the oscillation change with the strength of the interaction force. The determination of the tip–sample interaction force is a major issue in dynamic AFM because the force gives access to the materials properties of the sample; nonetheless the force is not a direct observable. Therefore, several methods have been proposed to reconstruct the force in dynamic AFM [12–18]. However, the use of force inversion methods has not been generalized in AM-AFM because the accuracy of some of the above methods is still under study. On the other hand, numerical simulations have been used to determine the maximum repulsive interaction forces, which are referred to as peak forces hereafter [19–23]. An analytical scaling law has been deduced to calculate the peak forces in air [21]. This method has been applied to determine the force on viral capsids in liquid [24]. However, the above expressions are often constrained to a specific interaction force model, such as Hertzian mechanics and thus their application range is somehow limited.

Numerical simulations have supported the development of AM-AFM by predicting several properties of the tip motion [25–27]. Those simulations provide the standards against which new experimental or analytical methods should be compared [28]. Recently, we have provided a broader numerical insight into the interaction forces in AM-AFM [19] by considering elastic, viscoelastic, electrostatic double layer and van der Waals interactions.

Here, we perform an extensive computational study of AM-AFM to obtain the peak forces of soft (50 MPa) and relatively rigid (2 GPa) materials for two different models of contact mechanics, namely Hertz [29] and Tatara [30–32]. We also provide a comparison between the numerical simulations and three analytical expressions [21,33–34]. The dependence of the peak force on a wide range of tip–microcantilever properties, operational parameters and mechanical properties of the sample is analyzed. The Young modulus (*E*_s_) ranges from 25 MPa to 2 GPa; the tip radius (*R*_t_) is varied between 5 and 10 nm; the free amplitude (*A*_0_) goes from 1 to 10 nm and the set-point amplitude (*A*_sp_) is within the 0.65*A*_0_ to 0.95*A*_0_ range.

The numerical results are compared to three analytical models, the parametrized [21], the average [33] and the linear one [34]. The numerical simulations show significant differences from the results given by the analytical approximations, although the parametrized expression is in good agreement with the Hertzian mechanics. The average model follows the trend of the Tatara model for the peak forces when varying the set-point amplitude for soft samples. For soft materials, the indentation of the tip could be higher than *A*_sp_. Thus the tip and the sample are in permanent contact during the whole oscillation cycle. In fact, the ability of exerting small forces and imaging materials in a non-invasive manner can be jeopardized because of the effect of a static deflection component when *A*_sp_/*A*_0_ decreases. We have also studied the relationship among peak forces, lateral resolution and sample properties for soft (50 MPa) and rigid (2 GPa) samples. We deduce a rule to image soft materials with a lateral resolution below 3 nm that involves the application of forces in the sub-100 pN regime, the use of cantilevers with force constants below 0.1 N/m, free amplitudes below 2 nm and relative sharp tips (*R*_t_ ≤ 5nm). AM-AFM operation at relatively high amplitudes can also lead to tip blunting [35–36]. The estimation of the peak force prior to performing the experiment could prevent tip damage.

## Results and Discussion

### Tip motion and contact time for soft and relatively rigid materials

In AM-AFM the equation of motion for the microcantilever–tip system is approximated by using the point-mass model [25],

[1]
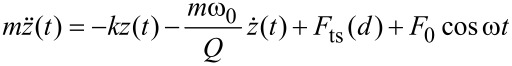


where *m* is the effective cantilever mass that includes the added mass of the fluid, and ω_0_, *Q*, *k* and *F*_ts_ are, respectively, angular resonant frequency, quality factor, spring constant and tip–sample interaction force. The point-mass model is suitable if the contribution of higher modes to the cantilever motion is negligible [37]. This could be the case in liquid for small free amplitudes, say below 1.5 nm [38]. At higher amplitudes, the tip–surface force generates higher harmonics components, which could lead to the momentary excitation of higher eigenmodes, in particular the second eigenmode [7]. To account for those effects we also describe the microcantilever–tip system by using an extended Euler–Bernoulli equation [39]. This model considers the cantilever as a continuous and uniform rectangular beam under the action of external forces,

[2]
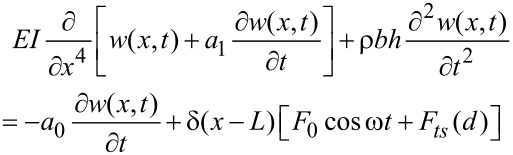


where *E* is the Young modulus of the cantilever, *I* the area moment of inertia, *a*_1_ the internal damping coefficient, ρ the mass density; *b*, *h* and *L* are, respectively, the width, height and length of the cantilever; *a*_0_ is the hydrodynamic damping; *w*(*x*,*t*) is the time dependent vertical displacement of the differential element of the beam placed at the *x* position, and *F*_ts_ tip–sample interaction force.

Equations 1 and 2 are numerically solved by using a fourth-order Runge–Kutta algorithm [40]. One should note that the use of Equations 1 and 2 in environments of low *Q* are valid for directly excited cantilevers, such as magnetic [41–43] or photothermal excitations [44–45]. The tip–sample interaction forces are modelled by using two different contact mechanics models, Hertz [29] and Tatara [30–32]. The widely used Hertz model gives the force as

[3]
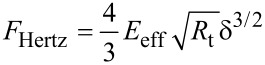


The Tatara contact mechanics has two key differences with respect to Hertzian mechanics. First it includes the finite size of the sample and second it also considers that the sample deformation happens symmetrically at both the tip–sample and the sample–substrate interfaces. Thus the vertical and lateral displacements are part of the contact force computed with this model.

[4]
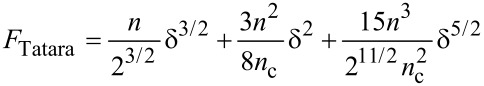


where

[5]
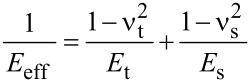


[6]
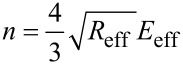


[7]
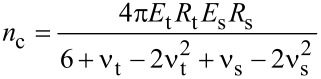


[8]
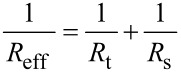


The subindexes “t” and “s” stand, respectively, for tip and sample. In the above equations, δ is the indentation depth, ν is the Poisson coefficient (ν_t_ = 0.3 and ν_s_ = 0.4) and *E* the Young modulus with *E*_t_ = 170 GPa. Each contact mechanics model is particularly suited for some types of deformations or strains. Hertz contact mechanics is used to deal with small deformations at the region of contact between tip and sample. Large deformations and finite object sizes are not well described by Hertz contact mechanics [32]. As a consequence, the Tatara model allows a maximum vertical deformation equal to *R*_eff_ and is particularly suited to describe large deformations (with respect to the original size) of relatively soft matter, in which a vertical force generates both vertical and lateral deformations. To apply contact mechanics models in conditions that do not meet the model assumptions will lead to unadequate numerical estimations.

We have not found significant differences in the calculation of peak forces by using the point-mass model and the continuous beam for free amplitudes below 2 nm. For that reason, the data for *A*_0_ = 1 nm has been obtained with the point-mass model while for *A*_0_ = 10 nm we have used the extended Euler–Bernoulli model.

**Figure 1:**
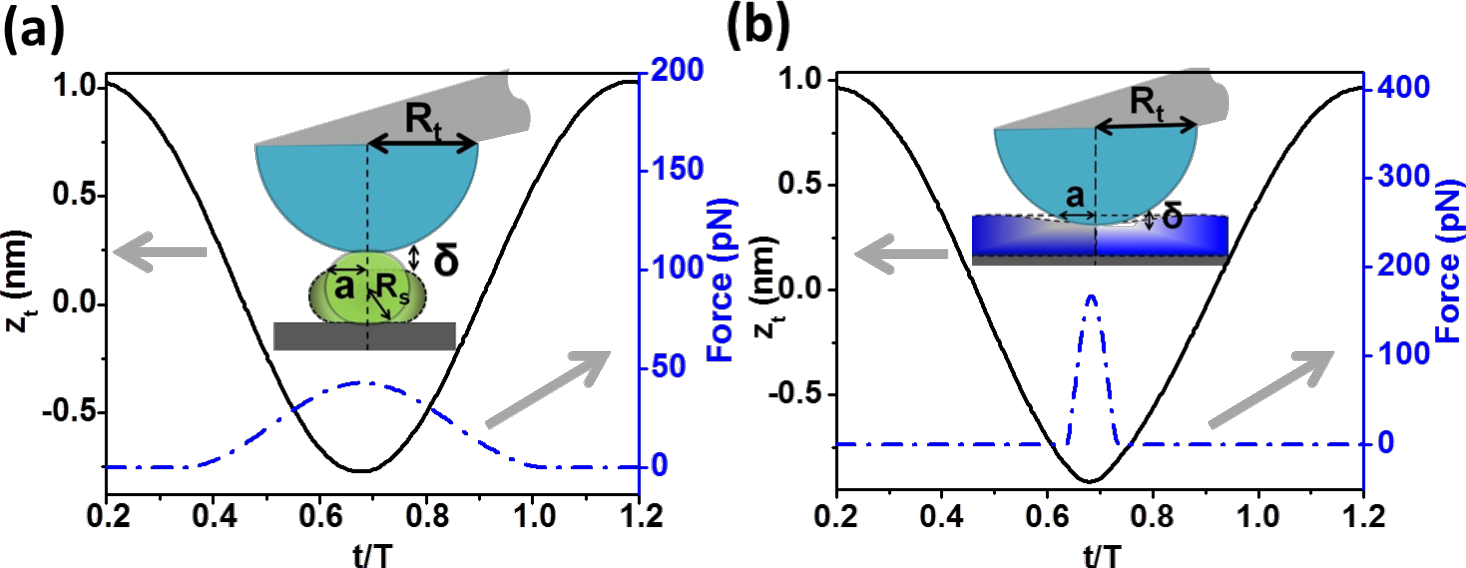
Peak forces, tip motion and contact time for two materials. (a) Soft sample (*E*_s_ = 50 MPa) simulated with the Tatara interaction force (Equation 4). (b) Rigid sample (*E*_s_ = 2 GPa) simulated with the Hertz interaction force (Equation 3). Simulation inputs: *k* = 0.1 N/m, *f*_0_ = 25 kHz, *Q* = 2, *A*_0_ = 1 nm, *R*_t_ = 5 nm, *R*_s_ = 4 nm and *A*_sp_ = 0.9 *A*_0_.

Figure 1 shows one period of the tip oscillation and the corresponding force. The peak force is defined as the maximum force point in the dashed line curves. The curves show a purely repulsive interaction, which starts as soon as the mechanical contact is established. The tip–sample interface according to Tatara (Figure 1a) or Hertz (Figure 1b) is also shown. Both contact mechancis models have been applied to describe the response of soft (50 MPa) and relatively rigid (2 GPa) surfaces. Figure S1 of Supporting Information File 1 shows the instantaneous force for a variable set-point amplitude.

### Simulated and analytical peak forces values: Dependence on the Young modulus and the set-point amplitude

Hu and Raman [21], Kowalewski and Legleiter [34] and Rodriguez and Garcia [33] have derived some analytical scaling laws to determine the interaction forces in AM-AFM. Hu and Raman parametrized the peak force (repulsive) by using a nonlinear asymptotic theory [46] and Hertz contact mechanics,

[9]
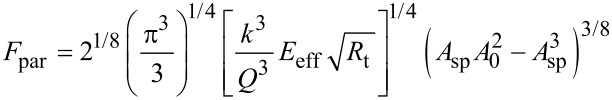


Rodriguez and Garcia, by using the virial-dissipation method [39,47–48], deduced the following expression for the mean value of the force during an oscillation,

[10]
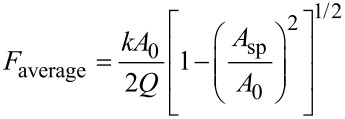


In the absence of long-range attractive forces, the average force can provide an estimation of the peak force.

Kowalewski and Legleiter proposed an extension of the Hooke law to determine the force in AM-AFM [34]. In this expression the force depends linearly on the amplitude reduction,

[11]
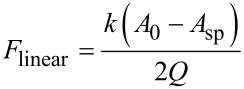


**Figure 2:**
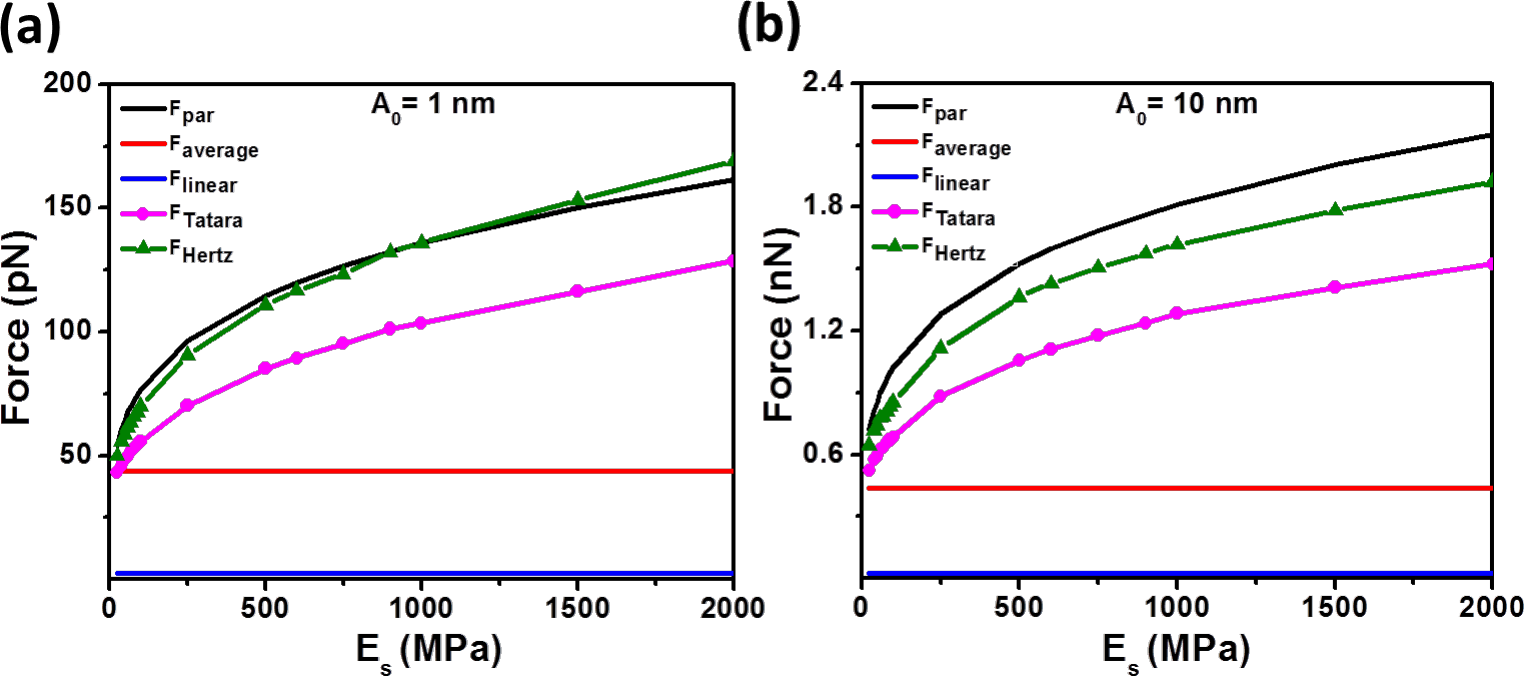
Peak force dependence on the Young modulus of the sample for different numerical simulations (Hertz and Tatara) and force analytical approximations (parametrized, average and linear). (a) *A*_0_ = 1 nm, *R*_t_ = 5 nm and (b) *A*_0_ = 10 nm, *R*_t_ = 5 nm. Other simulation inputs are: *k* = 0.1 N/m, *f_0_* = 25 kHz, *Q* = 2, *R*_s_ = 4 nm, and *A*_sp_ = 0.9 *A*_0_.

Figure 2 shows a comparison between the analytical expressions in Equations 9–11 and the numerical results (Hertz and Tatara). The comparison covers a range of the Young modulus from 25 to 2000 MPa. Hu and Raman parametrized force matches the numerical results derived from the Hertz model in some conditions. The parametrized model overestimates the peak forces in the case of very soft materials with a maximum error of 11%. On the other hand, for stiffer materials the force is underestimated with a maximum deviation of 16%. Numerical simulations performed with the Tatara model give smaller peak force values than those obtained from the Hertz model [19,49]. This is because in Tatara contact mechanics the deformation happens at both the tip–sample and the sample–substrate interfaces. The linear and average expressions fail to capture the trend of the numerical simulations because those expressions have been exclusively deduced from the dynamic properties of the tip motion and do not consider any influence of the materials properties of the sample. Additional comparisons by varying the tip radius are presented in Figure S2 of Supporting Information File 1.

**Figure 3:**
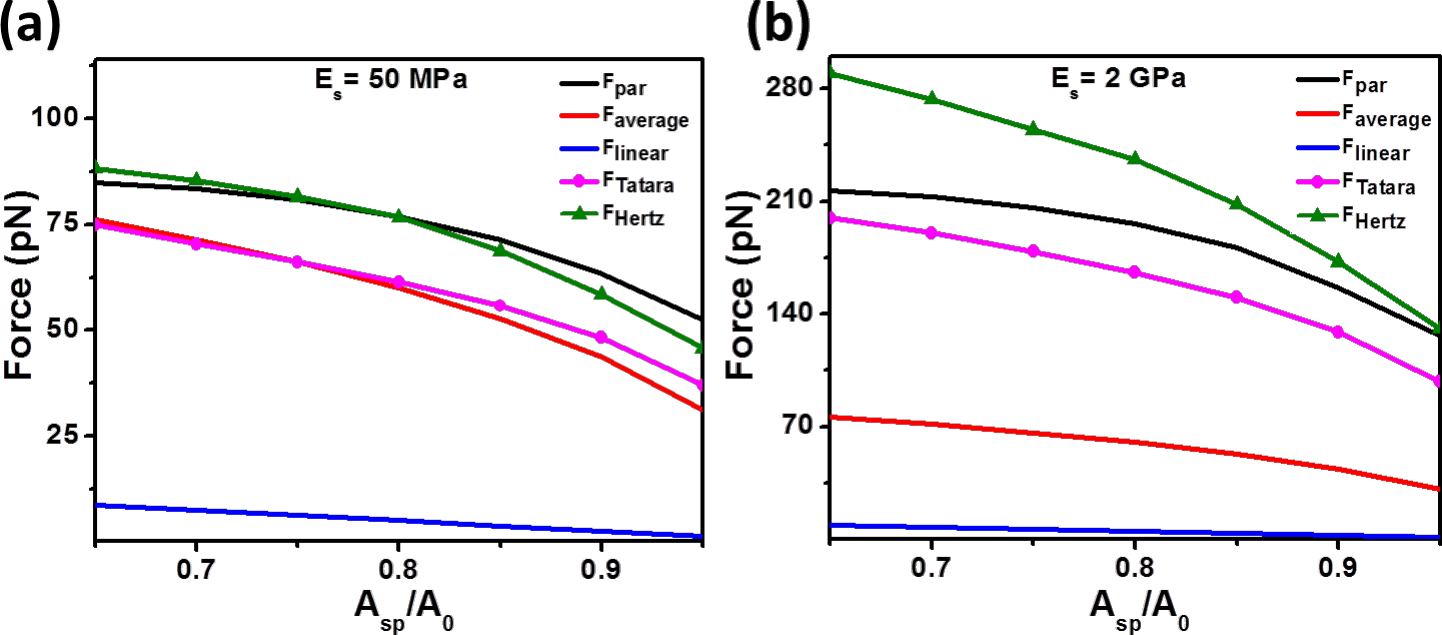
Dependence of the peak force on the set-point amplitude for different numerical simulations (Hertz and Tatara) and force analytical approximations (parametrized, average and linear). (a) *A*_0_ = 1 nm and *E*_s_ = 50 MPa. (b) *A*_0_ = 1 nm and *E*_s_ = 2 GPa. Simulation inputs are: *k* = 0.1 N/m, *f_0_* = 25 kHz, *Q* = 2, *R*_t_ = 5 nm, and *R*_s_ = 4 nm.

In Figure 3, the reduction of *A*_sp_ from 0.95*A*_0_ to 0.65*A*_0_ produces an increase of the peak force. This trend is reported by all the approximations and simulations. However, the linear approximation give values that are smaller by a factor of 5–100 compared with the numerical simulations. As a consequence, the linear approximation should not be used to estimate the peak force in AM-AFM. The average model gives values close to the Tatara model for soft materials (25–50 MPa). However, it fails to reproduce the data for stiffer surfaces. The average model gives the mean value of the forces, attractive and repulsive, acting on the tip during an oscillation period. Consequently, whenever the forces change significantly with the distance (stiff materials) this approximation will fail to give a good estimation of the peak force. The parametrized model gives a good numerical description of the peak forces derived from the Hertz model for relatively soft materials.

### Sample deformation in terms of *E*_s_, *A*_0_, and *R*_t_

**Figure 4:**
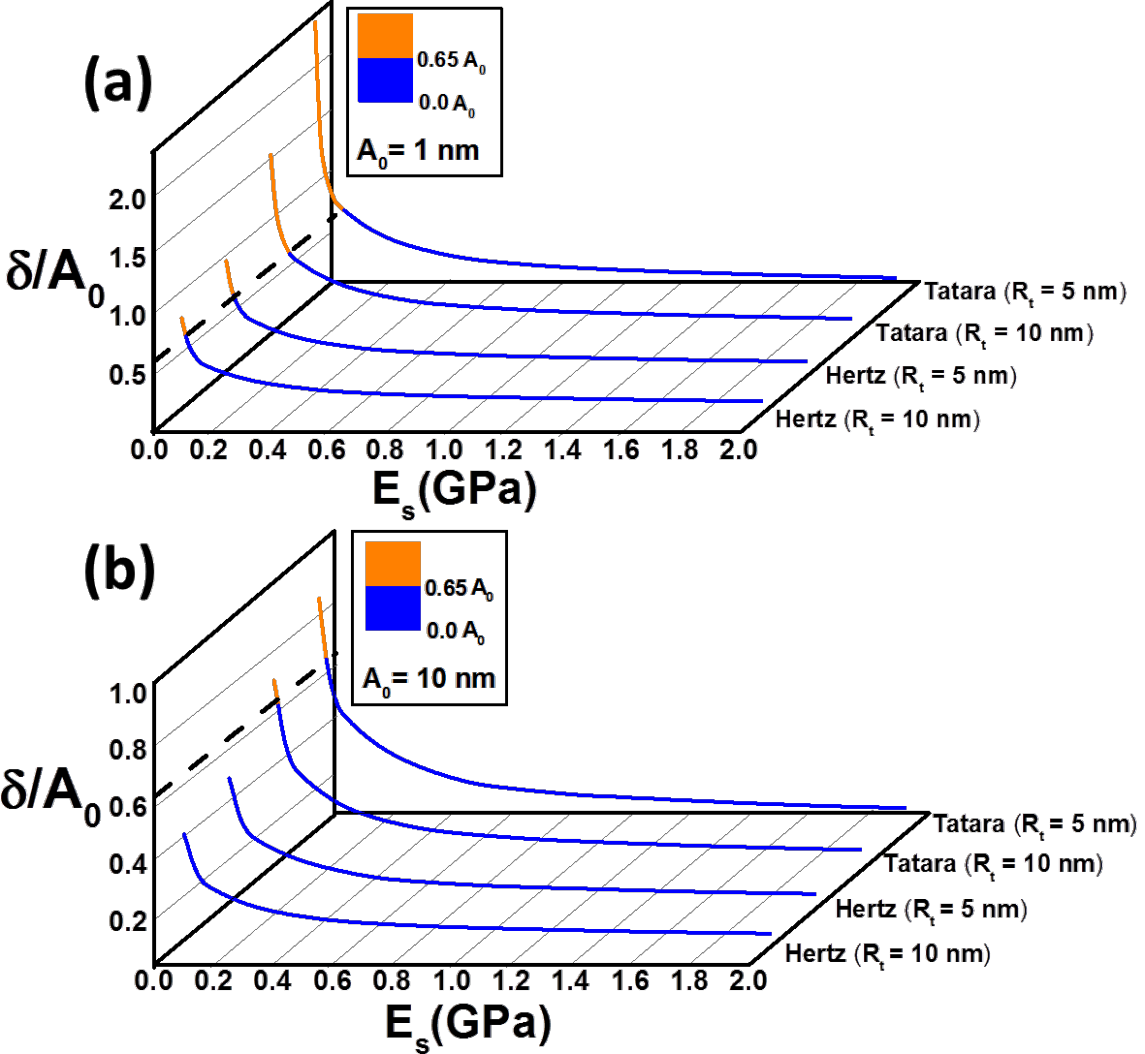
Normalized indentation as a function of the Young modulus of the sample for the Hertz and Tatara models. The indentation was normalized to the set-point amplitude: (a) *A*_0_ = 1 nm and (b) *A*_0_ = 10 nm. In the data above the dashed line indicates the point from which the whole oscillation is performed in contact to the material. Simulation inputs: *k* = 0.1 N/m, *f_0_* = 25 kHz, *Q* = 2, *A*_sp_ = 0.65*A*_0_, *R*_s_ = 4 nm, and two different *R*_t_ of 5 and 10 nm, respectively.

The deformation (indentation) exerted by the tip can be considered as an indicator of the degree of invasiveness of the technique. The dependence of the indentation on the Young modulus for Hertz and Tatara models is shown in Figure 4. The indentation values are computed and normalized by the free amplitude for two values, 1 and 10 nm, and two tip radii, 5 and 10 nm, respectively. The two-colour curve separates the operational parameters in which the deformation is smaller than the set-point amplitude from those in which the deformation is larger. As expected, the indentation increases by decreasing the Young modulus of the sample. Remarkably, for soft materials (i.e., those with *E*_s_ < 100 MPa) the indentation values are close to or even larger than the set-point amplitude. This means that the tip and the sample are in permanent contact during the whole oscillation. This result was observed experimentally by Raman et al. [6] while imaging cells. Hertz contact mechanics gives smaller indentations than Tatara. In addition, these results underline the relevance of the contribution from the static deflection, which cannot be neglected in liquid while imaging soft materials [19]. However, the above effect decreases, for the same ratio *A*_sp_/*A*_0_, when increasing the free amplitude as shown in Figure 4b.

### Lateral resolution at small peak forces

**Figure 5:**
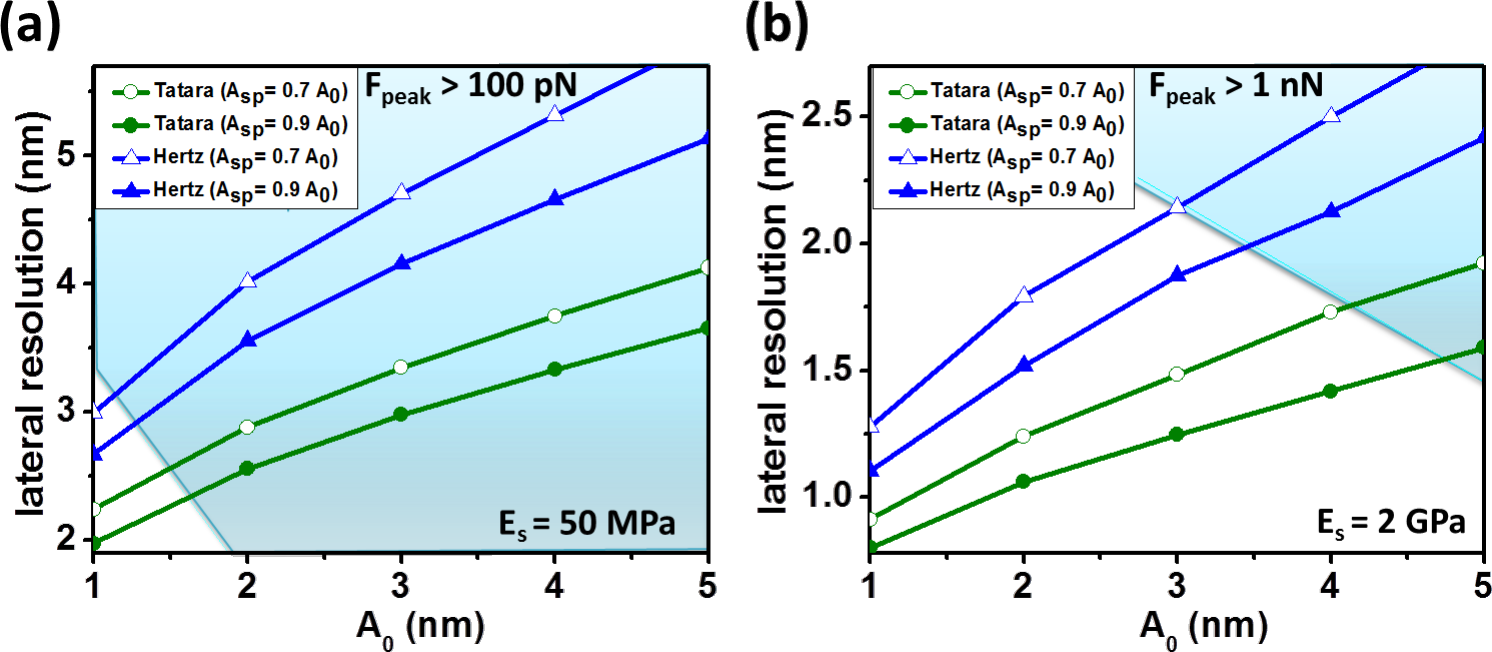
Lateral resolution maps for Hertz and Tatara contact mechanics. (a) *E*_s_ = 50 MPa. (b) *E*_s_ = 2 GPa. Filled symbols for *A*_sp_ = 0.9*A*_0_; empty symbols for *A*_sp_ = 0.7*A*_0_. Circles for Tatara and triangles for Hertz. Simulation inputs: *k* = 0.1 N/m, *f_0_* = 25 kHz, *Q* = 2, *A*_sp_ = 0.65*A*_0_, *R*_t_ = 5 nm, *R*_s_ = 4 nm, and two different *A*_sp_ of 0.9 and 0.7*A*_0_, respectively.

Imaging at high-spatial resolution demands a compromise between probe, operational parameters and sample properties. Figure 5 shows the lateral resolution as given by Hertz and Tatara models for two materials, respectively, *E*_s_ = 50 MPa and 2 GPa. The lateral resolution is defined as the contact diameter between tip and sample. In Figure 5 we visualize the interplay between the free oscillation amplitude and set-point amplitude with the lateral resolution and peak forces. For a fixed *A*_sp_/*A*_0_ ratio the contact diameter increases with *A*_0_, which reduces the lateral resolution. Lowering the *A*_sp_/*A*_0_ ratio down to the range between 0.65 and 0.95 also reduces the lateral resolution. In any situation the Tatara model gives a better lateral resolution than the Hertz model. This result can be traced back to the observation that, for the same operational conditions and probe values, the Tatara model gives smaller peak forces than the Hertz model. The lateral resolution also depends on the elastic response of the sample. As a general rule, the stiffer the sample the better the lateral resolution. Sub-nanometric resolution can be achieved by using small *A*_0_ and maintaining a relatively high *A*_sp_/*A*_0_ ratio for soft materials and rigid materials. It has been reported that, in some special situations, also for low *A*_sp_/*A*_0_ ratios a high resolution can obtained experimentally [28,50]. We note that for soft materials (*E*_s_ = 50 MPa) and in the best case scenario (Tatara model) a lateral resolution below 1 nm could only be reached by using a free amplitude below 0.5 nm. We have separated the plots into regions, for soft materials (Figure 5a) a small sub-100 pN force value is used; while for rigid materials (Figure 5b) a sub-1 nN reference value is considered.

## Conclusion

The numerical simulation of the tip motion in amplitude modulation AFM provides a comprehensive description of the factors that control the peak force and the lateral resolution in liquid. We have simulated the peak force for two contact mechanics models, Tatara and Hertz, and we have calculated three analytical approximations, linear, average and parametrized. The linear approximation fails to describe qualitatively and quantitatively the peak forces. The average model captures the peak force behaviour with the operational parameters but the quantitative agreement is poor. The parametrized model resembles the results given by Hertz for soft materials and small free amplitudes but its quantitative accuracy decreases by increasing the Young modulus. The results show that the discrepancy between the analytical and calculated values tends to decrease with smaller Young moduli and higher ratio *A*_sp_/*A*_0_. The spatial resolution depends on the operational parameters, the elastic response of the sample, the peak force, and the contact mechanics model. The conditions to achieve a high spatial resolution become more demanding for lower Young moduli of the samples. A high spatial resolution in liquid requires the use of rather small oscillation amplitudes. Sub-1 nm lateral resolutions for a soft material of a Young modulus of 50 MPa will require the use of a free amplitude of 0.5 nm or less. Lowering the free amplitude of the oscillation improves the lateral resolution in liquid. The resolution increases in line with the Young modulus of the sample, while keeping the operational parameters constant. The lateral resolution depends on the contact mechanics model used to characterize the sample deformation. In the Tatara model the sample is finite, consequently the stress is relaxed both vertically and laterally, which, for the same indentation, provides smaller forces and consequently a better resolution that the result given by Hertz model.

The results presented here provide a good estimation of the peak force values experienced by the samples observed with an AFM in liquid. However, the simulations have been performed without considering hydration layers or viscoelastic effects that arise either from the sample or the hydration layer. Those effects could modify the peak force values reported here, although we do not expect significant changes for the data acquired under the conditions for a high spatial resolution (sub-5 nm).

## Supporting Information

File 1 Additional experimental details.
