# Supplementary material for: Peak forces and lateral resolution in amplitude modulation force microscopy in liquid
Source: Beilstein J Nanotechnol. 2013 Dec 6;4:852–9. doi: 10.3762/bjnano.4.96 (PMC3869265; doi:10.3762/bjnano.4.96)
Supplement: File 1 — Additional experimental details. [file Beilstein_J_Nanotechnol-04-852-s001.pdf]

Supporting Information

for

Peak forces and lateral resolution in amplitude modulation force microscopy in liquid

Horacio V. Guzman and Ricardo Garcia*

Address: Instituto de Ciencia de Materiales de Madrid, CSIC, Sor Juan Ines de la Cruz 3, 28049 Madrid, Spain

Email: Ricardo Garcia* - r.garcia@csic.es

* Corresponding author

Additional simulation details

Tip motion and contact time for soft and rigid materials while varying the set-point amplitude

Figure S1 shows the numerical simulations of the force, contact time and the tip motion as given by Hertz and Tatara models, for rigid (2 GPa) and soft (50MPa) materials. The curves show the data for two set-point amplitudes (A_{sp}) within an oscillation cycle at the steady state regime. There is a strong influence of the Young modulus on the contact time (t_c) and the force as the $\frac{A_{sp}}{A_0}$ ratio is decreased.

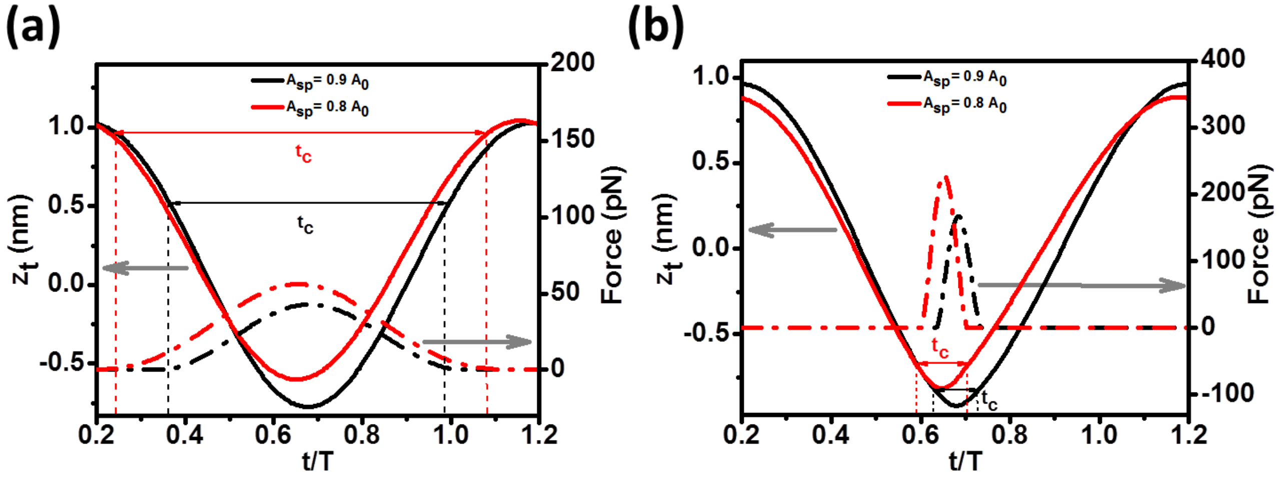

Figure S1: Peak force, tip motion and contact time for two materials. (a) Soft sample (E_s=50 MPa) simulated with the Tatara model. (b) Rigid sample (E_s=2 GPa) simulated with the Hertz model. Simulation inputs: $k = 0.1$ N/m, $f_0 = 25$ kHz, $Q = 2$, $A_0 = 1$ nm, $R_t = 5$ nm, $R_s = 4$ nm and two different A_{sp}, $0.9A_0$ and $0.8A_0$.

Simulations and analytical peak forces. Dependence on the Young modulus, free amplitude and tip radius

Figure S2 shows a comparison of the peak force obtained with the analytical expressions Equations 8–10 of the main text and the numerical results (Hertz and Tatara) over a Young modulus range from 25 to 2000 MPa. We also study the influence of the tip radius on the peak force. The peak force increase with the radius, however, the increase is much smaller than what could be expected from the expression of the force. The dependence with the free amplitude is more remarkable. By increasing the free amplitude (A_0) by a factor of almost 10, the peak force shows an increase of a factor of nearly 20. Note that the average and linear models do not depend on either the Young modulus or the tip radius.

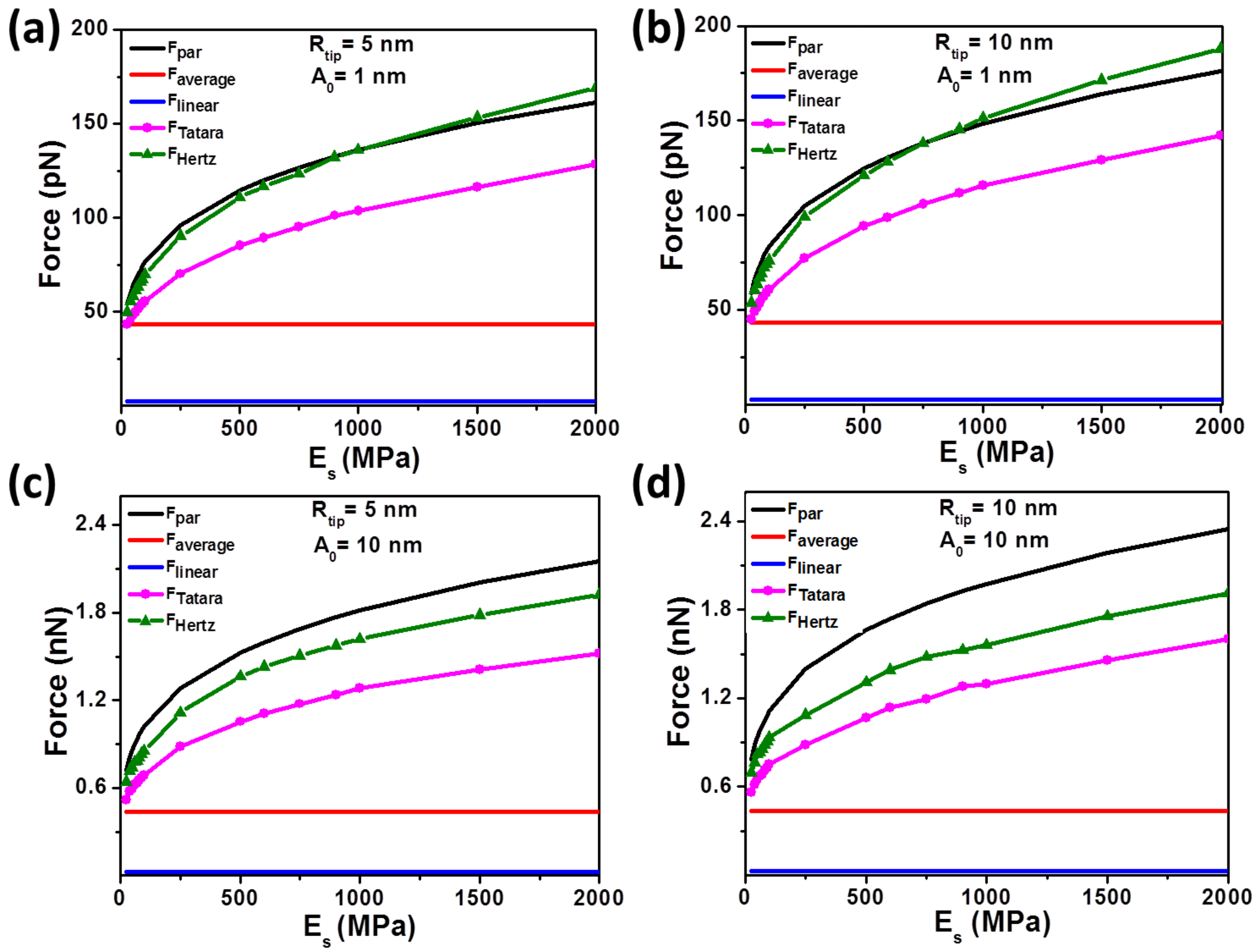

Figure S2: Peak force dependence on the sample Young modulus for different numerical simulations (Hertz and Tatara) and force approximations (parametrized, average and linear). (a) $A_0 = 1$ nm, $R_t = 5$ nm and (b) $A_0 = 1$ nm, $R_t = 10$ nm (c) $A_0 = 10$ nm, $R_t = 5$ nm and (d) $A_0 = 10$ nm, $R_t = 10$ nm. Other simulation inputs are: $k = 0.1$ N/m, $f_0 = 25$ kHz, $Q = 2$, $R_s = 4$ nm and $A_{sp} = 0.9A_0$.
